# Supplementary material for: Development of a High-Quality/Yield Long-Read Sequencing-Adaptable DNA Extraction Method for Crop Seeds
Source: Plants (Basel). 2023 Aug 17;12(16):2971. doi: 10.3390/plants12162971 (PMC10457885; doi:10.3390/plants12162971)

Table S1. Oxford Nanopore statistics for sequenced samples

	CTA B1[*1]	CTAB2 [*1]	IMP Boom1[*1]	IMP Boom 2[*1]	IMP Boom 3	IMP Boom 4	kit
Input DNA (ng) [*2]	1080	1000	999	1089	500	500	500
DNA concentration after End-prep[*3] (ng/μL)	8.04	9.32	13.60	16.10	10.40	39.40	26.4 0
Library concentration [*4] (ng/μL)	0.60	1.54	33.60	31.20	22.60	17.80	9.64
Total run time (h)	24	24	19.5	19.5	24	24	24
Number of pores (QC) [*5]	41	45	76	45	81	54	86
Number of pores immediately after sequencing [*6]	34	40	67	46	76	54	70
Read length N_{50} (kbp)	0.95	1.11	14.96	14.36	18.11	20.96	12.4 0
Mean read length (bp)	686	581	2,904	2,796	3,273	3,242	4,00 7
Median read length (bp)	356	271	709	618	719	664	1,18 0
Total reads (k reads)	14.85	96.9	94.13	38.79	85.07	66.52	165. 81
Sequencing yield (Mbp)	9.68	73.76	325.04	116.60	300.09	226.60	732. 09

For both the CTAB and IMP Boom methods, two long-read sequencing runs were performed, but only one for kit due to low DNA yield. To obtain sufficient DNA for a DNA library in the kit method, three DNA tubes were pooled together. The DNA libraries of IMP Boom3, 4, and kit were constructed using intact DNA.

[*1] These libraries were constructed using DNA fragmented by g-TUBEs.

[*2] The amount of genomic DNA for library construction.

[*3] The concentration of DNA eluted in nuclease-free water after end-pred reaction.

[*4] The concentration of the library used for sequencing.

[*5] The number of pores obtained during the pre-sequencing quality control (QC) process.

[*6] The number of pores immediately after the start of sequencing.

Table S2. Nucleotide sequences of primers used in this study

Primers	Forward	Reverse
For quantification of nuclear, chloroplast, and mitochondrial DNA via qPCR		
Nuclear DNA (GmTubline)	GAGAAGAGTATCCGGATAGG	GAGCTTGAGTGTTCGGAAAC
Plastid DNA	CTTCTACAACCCCTGATATTCAAAG	ACATACATAATAAGATGTGAATGATAC
Mitochondrial DNA	AAAGCTAAAAGTAGGCTCGCTATTG	TAAGTCAAGTACTACACGAGCGAAG
For genotyping based on SSR markers		
Satt181	TGGCTAGCAGATTGACA	GGAGCATAGCTGTTAGGA
Satt373	TCCGCGAGATAAATTCGTAAAAT	GGCCAGATACCCAAGTTGTACTTGT
For PCR of target genes		
GmActin	ACCTGTACGGTAACATTGTCCTTTC	GAATCTTTGATTAGAGAGCTTGTGC
GmSACPD-B	ACACTTCATTTCAAGAGAGGGCAAC	TCTCACATGTTAACGTAGCGTATAC
GmSACPD-A	ACACTTCATTTCAAGAGAGGGCAAC	AAATAACAAGCCATATCCCAGCAAG
GmSACPD-C	ACACGTCATTCCAAGAGCGAGCAAC	CGTACTCAAAATGCCACCTACAAAC

Table S3. Costs of reagents and kits used in this study

Reagents or kit	Company	Volume	No.	Cost (Japanese Yen)	Cost (US dollar)
Reagents purchased					
Guanidine thiocyanate	Nakarai Tesque	1 kg	17345-51	¥31,100	
PVPP (Polyvinylpolypyrrolidone)	Sigma–Aldrich	1 kg	77627	¥78,200	
Diatomaceous earth	Sigma–Aldrich	1 kg	D3877	¥14,800	
Triton X-100	Nakarai Tesque	500 mL	12969-25	¥2,650	
Tween-20	Nakarai Tesque	500 mL	28353-85	¥3,350	
Reagents created					
Guanidine thiocyanate stock	-	1 L		¥22,261	
Lysis buffer	-	1 L		¥3,715	
Binding buffer	-	480 mL		¥8,926	
Wash buffer	-	500 mL		¥5,565	
Per sample of optimized Boom method					
Lysis buffer	-	6 mL		¥22	
Binding buffer	-	10 mL		¥186	
Wash buffer	-	1.5 mL		¥56	
Total				¥264	US$2.0
DNeasy Plant Mini kit (50)	Qiagen	50 times	69104	¥32,000	
Per sample				¥640	US$4.8
Genomic DNA Extraction kit Mini (Plant)	RBC Bioscience	100 times	YGP100	¥30,000	
Per sample				¥300	US$2.3

Figure S1. Comparison of read length frequencies of DNA extracted using the kit, IMP Boom, and CTAB methods. The read length N50 values for each library are shown in the upper-right corner of the figure. Purple, blue, and black arrows indicate maximum read lengths for the Boom3, 4, CTAB1, 2, and kit methods, respectively.

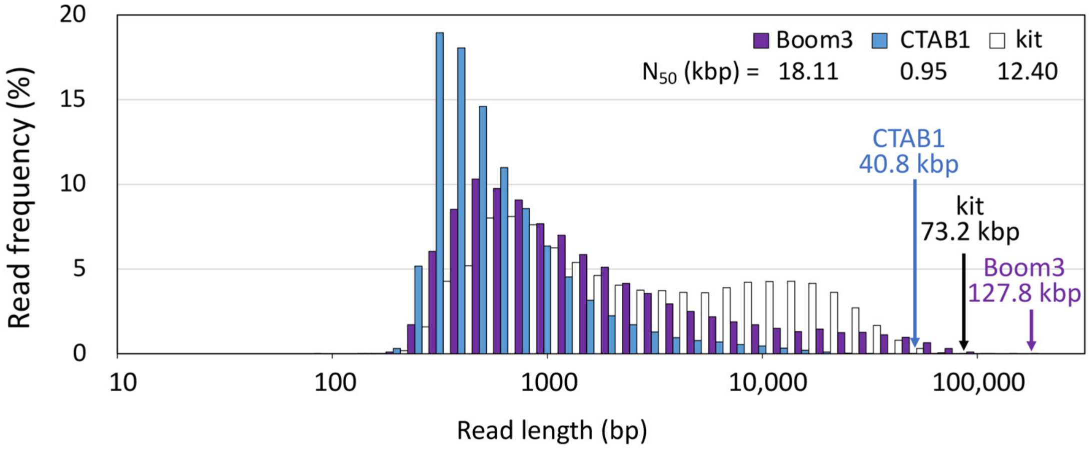

Figure S2. Versatility of DNA obtained using the IMP Boom method. (A) Electrophoresis of SSR markers on a 3% agarose gel. Genotyping for the Satt181 and Satt373 markers in the DNA extracted from RIL seeds of different genetic backgrounds. (B) Gene regions of GmActin, GmSACPD–B, GmSACPD–A, and GmSACPD–C were amplified using PCR from DNA extracted from Enrei seeds. SM is a size marker containing DNA lengths from 100 to 10,000 bp.

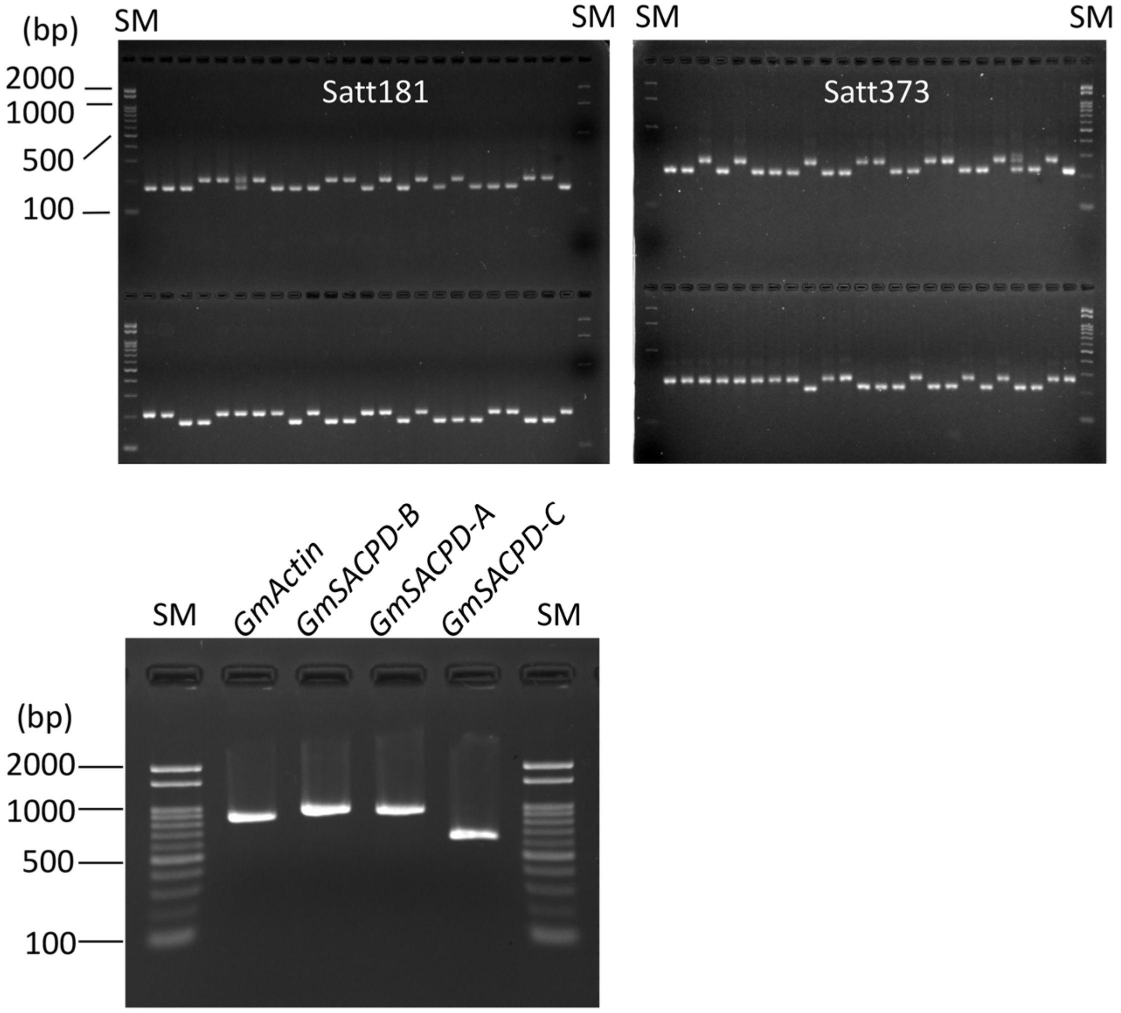

Supplement: Supplementary file 1 [file plants-12-02971-s001.zip › plants-2444397-supplementary.pdf]
